# High potential thermoelectric figure of merit in ternary La_3_Cu_3_X_4_ (X = P, As, Sb and Bi) compounds

**DOI:** 10.1038/s41598-017-14658-5

**Published:** 2017-10-27

**Authors:** Tribhuwan Pandey, David S. Parker

**Affiliations:** Materials Science and Technology Division, Oak Ridge National Laboratory, Oak Ridge, Tennessee 37831 USA

## Abstract

We investigate the thermoelectric properties of the relatively unexplored rare-earth ternary compounds La_3_Cu_3_X_4_ (X = Bi, Sb, As, and P) using first principles electronic structure and Boltzmann transport calculations. These compounds, of which the La_3_Cu_3_Sb_4_ and La_3_Cu_3_Bi_4_ have previously been synthesized, are all predicted to be semiconductors and present a wide range of bandgaps varying from 0.24 eV (for the Bi compound) to 0.87 eV (for the P compound). We further find a mixture of light and heavy bands, which results in a high thermoelectric power factor. In addition, as discussed in our previous study (Phys. Rev. B 95 (22), 224306, 2017) at high temperatures of 1000 K these compounds exhibit lattice thermal conductivity less than 1 W/mK. The combination of low thermal conductivity and good transport properties results in a predicted *ZT* as high as ~1.5 for both La_3_Cu_3_P_4_ and La_3_Cu_3_As_4_, under high p-type doping. This predicted high performance makes these compounds promising candidates for high temperature thermoelectric applications and thus merits further experimental investigation.

## Introduction

Thermoelectric materials can convert waste heat to useful electricity. The performance of a thermoelectric material is determined by the figure of merit (*ZT*) given by $\frac{{S}^{2}\sigma }{\kappa }$, where S is the thermopower, *σ* is the electrical conductivity, and *κ* is the thermal conductivity. The quantity *S* ^2^ *σ* is referred as the power factor. Over the last few decades both theoretical and experimental research on thermoelectrics has escalated due to the development of new synthesis techniques, as well as theoretical models. Favorable thermoelectric properties have been realized in several materials such as inorganic clathrates^1,2^, skutterudites^3–5^, magnesium group IV compounds^6–8^, half Heusler alloys^9–11^, SnSe^12–14^, and PbTe^15–17^. Additionally nanostructuring, which reduces the lattice thermal conductivity *via* enhanced phonon scattering can provide a route to achieve high *ZT*. Various theoretical and experimental studies have discussed the role of mass anisotropy^14,16,18^, quantum confinement^19,20^, and band structure engineering^14,21–23^ in designing materials with enhanced thermoelectric properties.

Mahan and Sofo proposed two decades ago that a delta-function electron density of states distribution near the band edge results in large thermopower^24^. This delta function behavior in the density of states can be realized in nature by the *f*-electron level of the rare earth element in a material^25,26^. For example, the intermetallic alloy YbAl_3_, exhibits highest power factor among all known thermoelectric materials in the temperature range 100–300 K^27,28^. This large factor is attributed to a sharp feature in the density of states near the band edge, which originates from the interaction of Yb 4*f* electrons with the conduction electrons. However, due to a large electronic thermal conductivity (*κ*_*e*_) only a small *ZT* of 0.13 was achieved^28^. On the contrary *κ*_*e*_ can be strongly suppressed in semiconducting rare earth compounds and more importantly they present the possibility to engender not only large power factor but also high *ZT*.

While some study of ternary rare earth semiconducting compounds aimed at thermoelectric applications has been carried out^29–31^ a detailed investigation of materials properties that are essential for high *ZT* is still elusive. In this paper, we present a comprehensive study of the thermoelectric properties of the relatively unexplored ternary La_3_Cu_3_X_4_ (X = P, As, Sb and Bi) compounds. As the bandgap of La_3_Cu_3_Sb_4_ and La_3_Cu_3_Bi_4_ is calculated to be < 0.33 eV, here we explore the possibilities of enhancing their bandgap by studying the homologous compounds such as La_3_Cu_3_P_4_ and La_3_Cu_3_As_4_. The latter two compounds are not known experimentally, so we asses their structural and thermodynamic stability by using structure prediction method. We found that the La_3_Cu_3_P_4_ and La_3_Cu_3_As_4_ also prefer the same crystal structure as the Bi and Sb counterparts. We find these compounds to be semiconducting with bandgaps ranging from 0.23 eV to 0.87 eV. In addition, these compounds exhibit structural and electronic features such as high band degeneracy, and combination of both heavy and light bands near the band edges, which yield high power factor. Furthermore as discussed in our recent study^32^, these compounds exhibit low lattice thermal conductivity (<1 W/mK) at high temperatures. By optimizing the *ZT* with respect to the doping concentrations at different temperatures, we find a peak *ZT* value of ~1.5 for *p*-type La_3_Cu_3_P_4_ and La_3_Cu_3_As_4_ at 1000 K at doping concentration of 7 × 10^20^ cm^−3^.

## Results

### Crystal structure and thermodynamical stability of La_3_Cu_3_X_4_ (X = P, As, Sb, and Bi) compounds

**Figure 1:**
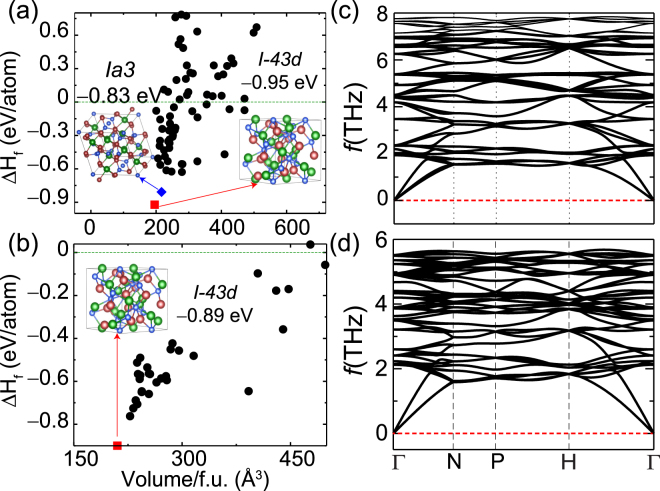
Formation enthalpies (ΔH_*f*_) for various configurations of (**a**) La_3_Cu_3_P_4_, and (**b**) La_3_Cu_3_As_4_ calculated with respect to elemental phases as a function of volume per formula unit. The structure with space group *I*-43*d* (red square) was found to be most stable for both La_3_Cu_3_P_4_ and La_3_Cu_3_As_4_. The 0 eV line in (**a**,**b**) denotes zero formation enthalpy. The phonon dispersion curve for La_3_Cu_3_P_4_, and La_3_Cu_3_As_4_ are shown in part (**c**,**d**), respectively. The absence of negative frequencies confirms the dynamical stability of these compounds.

Previous experimental reports have established that La_3_Cu_3_Sb_4_ and La_3_Cu_3_Bi_4_ crystallize in the cubic structure, with the space group *I*-*43d* ^33,34^. However, the P and As analogs are not yet reported, despite the existence of compounds with other stoichiometries, such as La_5_Cu_19_P_12_^35^, LaCu_4_P_3_^36^, LaCu_1.09_P_2_^36^, and LaCu_1.233_As_2_^37^ in these ternary groups. With this in mind, we made a detailed theoretical study of possible physical structure of the ternaries La_3_Cu_3_P_4_/La_3_Cu_3_As_4_ and their thermodynamical stability. First one must determine the likely *physical structure* of these ternary phases, since the thermoelectric properties are necessarily sensitive to the actual structure. This is a substantial task, but one for which powerful computational techniques have recently been developed^38–42^. Here to predict the structure of La_3_Cu_3_P_4_ and La_3_Cu_3_As_4_ we use a global structure search method with the particle swarm optimization (PSO)^41^ algorithm as implemented in the Calypso code^43^. Enthalpy of formation (Δ*H*_*f*_) for the prospective phases of La_3_Cu_3_P_4_ and La_3_Cu_3_As_4_ was calculated with respect to their elemental phases. The resulting energetic diagram is shown in Fig. 1(a,b) for La_3_Cu_3_P_4_ and La_3_Cu_3_As_4_, respectively. In the case of La_3_Cu_3_P_4_ we find two structures with space group *I*-*43d* and *Ia3* to be nearly equal in energy.

**Figure 2:**
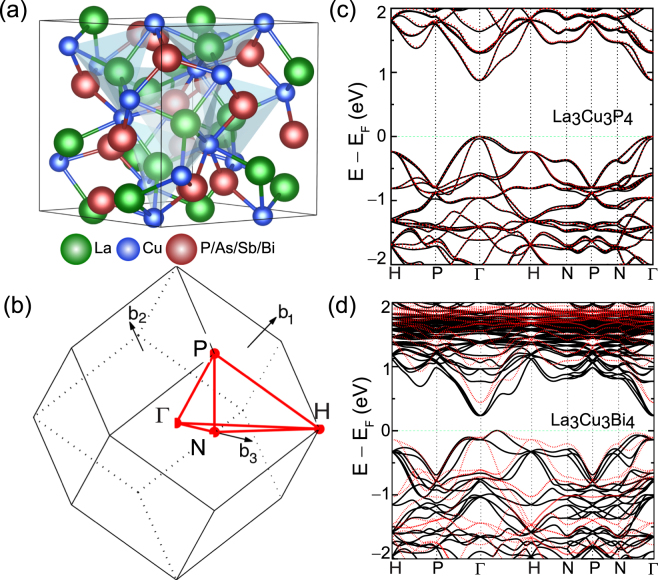
(**a**) Crystal structure, and (**b**) Brillouin zone for La_3_Cu_3_X_4_ (X = P, As, Sb and Bi). The green, blue, and red colors represent the La, Cu, and P/As/Sb/Bi atoms, respectively. Band structure as calculated with TB-mBJ functional for (**c**) La_3_Cu_3_P_4_, and (**d**) La_3_Cu_3_Bi_4_. The valence band maxima is referenced to 0 eV. The solid black lines represent band structure with spin orbit coupling and dotted red lines represent band structure without spin orbit coupling.

To further investigate their stability we present phonon calculations for both structures. An enlarged version of Fig. 1(c,d) and its effect of lattice thermal conductivity is already published and discussed at length in our recent study^32^. Here phonon dispersions are presented to address the dynamical stability of these compounds. As shown in Fig. 1(c) the structure with the space group *I*-*43d* is dynamically stable, whereas the structure with space group *Ia3*, is dynamically unstable (Supplementary Information Fig. S1). For La_3_Cu_3_As_4_ the structure with space group *I*-*43d* was found to be the most stable (Fig. 1(d)). This analysis suggests that the experimentally grown La_3_Cu_3_P_4_ and La_3_Cu_3_As_4_ would favor crystallizing in the *I*-*43d* space group, which is the same as the other homologous compounds La_3_Cu_3_Sb_4_ and La_3_Cu_3_Bi_4_. The computed DFT lattice parameters of all four compounds studied here are shown in Table 1. All the calculations presented in this work are done with these optimized lattice parameters. Because of the large electronegativity difference between La and X, La transfers its electrons to X atoms. Hence the structure can be described as a Cu-X tetrahedron where the La atoms are separated by Cu-X tetrahedra as shown in Fig. 2(a).

**Table 1:** The GGA functional optimized lattice parameters of La_3_Cu_3_X_4_ (X = P, As, Sb, and Bi) along with the available experimental lattice parameters and other calculated results.

System	*a*_*DFT*_ (Å)	*a*_*exp*_ (Å)	bandgap (eV)	${{\bf{m}}}_{{\boldsymbol{e}}}^{{\boldsymbol{\ast }}}$	${{\bf{m}}}_{{\boldsymbol{h}}}^{{\boldsymbol{\ast }}}$	Δ*E*_D_ (meV/atom)	*κ*_*latt*_ @ 300 K (W/mK)
La_3_Cu_3_ ${{\rm{P}}}_{4}^{\dagger }$	9.22	NA	0.88	0.56	1.38	190	1.31^32^
La_3_Cu_3_ ${{\rm{As}}}_{4}^{\dagger }$	9.44	NA	0.72	0.45	1.23	151	1.69^32^
La_3_Cu_3_Sb_4_	9.86	9.83^33^	0.33	0.32	0.85	93	3.35^32^
La_3_Cu_3_Bi_4_	10.06	9.97^33^	0.24	0.38	0.76	60	2.60^32^

The bandgap is calculated with TB-mBJ functional including the spin orbit coupling. To describe the thermodynamical stability, decomposition enthalpy (Δ*E*_D_) is also given in meV/atom for all four compounds. Additionally, the room temperature lattice thermal conductivity (*κ*_*latt*_) as calculated in ref.^32^ is also shown for comparison. Dagger(^†^), represents structures generated by particle swarm optimization (PSO) algorithm using the Calypso code.

Once the crystal structure of a material is known, next task is to explore its thermodynamical stability. To predict the thermodynamic stability finding a negative formation enthalpy for La_3_Cu_3_P_4_, and La_3_Cu_3_As_4_ with respect to their elemental phases is a very weak test of stability as most missing materials have negative formation enthalpies but are instead unstable with respect to other binary/ternary competing phases. Hence, one must verify that the compound is thermodynamically stable against decomposition, not only into the respective elements, but into *every other* possible set of constituents, including all binary and ternary phases. Thermodyamical stability of a compound is generally described by calculating its decomposition enthalpy (Δ*E*_D_) with respect to all other stable competing phases. Higher Δ*E*_D_ means less probability of a phase being thermodynamically stable.

For the ternary phase diagram^44^ of La-Cu-P and La-Cu-As, there are over *twenty* possible phases (these are listed and discussed in the Supplementary Information). We list there the formation enthalpy for all phases given by the Materials Project^45–47^ and AFLOWLIB data base^48,49^, as well as those we have directly calculated. Fortunately, nearly all of the binary compounds listed there have formation enthalpies substantially smaller in magnitude than La_3_Cu_3_P_4_ and La_3_Cu_3_As_4_, so that the most potential decomposition pathways are energetically blocked. The only exception to this for La_3_Cu_3_P_4_ are LaP and LaP_2_, which show a substantial formation enthalpy magnitude of −1.5 eV/atom and −1.1 eV/atom, respectively. When one assesses a potential decomposition of the prospective La_3_Cu_3_P_4_ into LaP and LaCu_4_P_3_, one then finds a marginal decomposition, with the energy gained being ~190 meV/atom. Similarly for La_3_Cu_3_As_4_; LaAs, La_4_As_3_ and LaAs_2_ show higher formation energy than La_3_Cu_3_As_4_. However as discussed in the Supplementary Information, on considering the decomposition of La_3_Cu_3_As_4_ into LaAs, La_4_As_3_ and LaAs_2_ *via* most favorable pathways the energy gain is only ~150 meV/atom. While this decomposition is possible, one often finds compounds with similar marginal calculated energetics which do in fact form, potentially in a metastable configuration. For example, these same calculations find the experimentally known (with melting points above 1000 K) La_3_Cu_3_Sb_4_ and La_3_Cu_3_Bi_4_ compounds to also be thermodynamically unstable, with decomposition enthalpies of 93 meV/atom and 60 meV/atom, respectively as shown in Table 1. In addition, an examination of published data (The Materials Project)^45,50^ finds several known compounds in the Copper-pnictogen binary phases with high decomposition enthalpies which nonetheless form. For example, Cu_3_As_4_^51^, Cu_3_N^52^, and Cu_3_Sb^53^ have calculated decomposition enthalpies of 264, 189 and 114 meV/atom, respectively. Furthermore, in a recent study on half Heusler alloys Butler *et*. *al* ^54^ showed that the majority of half-Heusler compounds which form experimentally show decomposition enthalpies in the range of 0–100 meV/atom. Given this we find there is a substantial probability that La_3_Cu_3_P_4_ and La_3_Cu_3_As_4_ will in fact form in the studied structure, and leave the matter for experimental inquiry. It is our opinion that the predicted high thermoelectric performance (described below) of this phase, if it exists, justifies experimental efforts to obtain it, despite the question about its thermodynamic stability.

### Electronic structure

**Figure 3:**
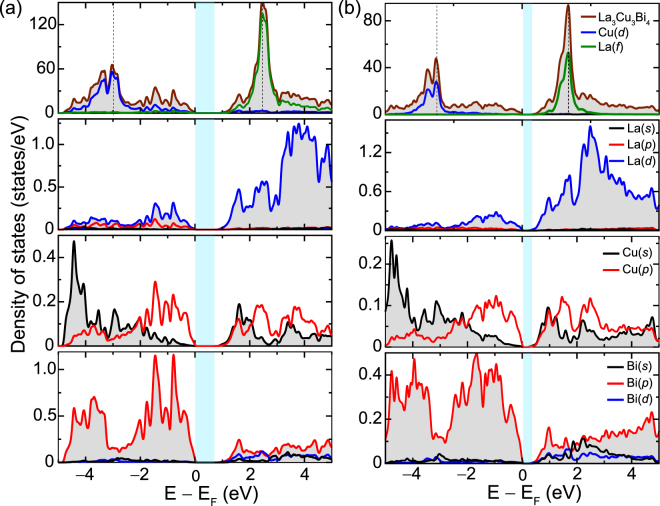
Total and partial density of states obtained using the TB-mBJ functional for (**a**) La_3_Cu_3_P_4_, and (**b**) La_3_Cu_3_Bi_4_. The valence band maxima is referenced to 0 eV. Overall DOS exhibits similar features for both compounds. Legends for (**a**) can be obtained by replacing Bi with P in (**b**).

We begin by analyzing the band structures of La_3_Cu_3_P_4_ and La_3_Cu_3_Bi_4_, depicted in Fig. 2(c,d). The band structures of La_3_Cu_3_As_4_ and La_3_Cu_3_Sb_4_ can be found in the Supplementary Information Fig. S2. Among the four compounds La_3_Cu_3_Sb_4_, and La_3_Cu_3_Bi_4_ are indirect bandgap semiconductors with valence band maximum (VBM) in between Γ-H direction and conduction band minimum (CBM) at Γ point. The band structures of La_3_Cu_3_Bi_4_ and La_3_Cu_3_Sb_4_ are similar to the other analogs compounds such as La_3_Au_3_Sb_4_ and La_3_Au_3_Bi_4_^55,56^. On the other hand La_3_Cu_3_P_4_ and La_3_Cu_3_As_4_ are direct gap semiconductors with both VBM and CBM at Γ point. The calculated bandgaps for La_3_Cu_3_P_4_, La_3_Cu_3_As_4_, La_3_Cu_3_Sb_4_, and La_3_Cu_3_Bi_4_ are 0.88, 0.72, 0.33, and 0.24 eV, respectively. The smaller bandgap of the Bi and Sb compounds suggest that bipolar conduction will be a concern with these materials, and therefore their thermoelectric performance will potentially be optimal at lower temperatures. The other two compounds, should be largely free from bipolar conduction even at high temperatures. The calculated density of states (DOS) for La_3_Cu_3_P_4_ and La_3_Cu_3_Bi_4_ are presented in Fig. 3(a,b), respectively. The DOS for La_3_Cu_3_As_4_ and La_3_Cu_3_Sb_4_ can be found in the Supplementary Information Fig. S3. From the DOS plots, one sees that while the valence bands for all four compounds have substantial X (X = P, As, Sb, and Bi) *p* character, the conduction bands have primarily Cu-*s*/*p* and Bi-*p* character (some contribution from La-*d* states can also be seen). The La-*f* and Cu-*d* states are located more than 2 eV away from the CBM and the VBM, respectively.

The most significant effect of the inclusion of spin orbit coupling is to reduce the calculated bandgap. The spin orbit coupling is rather important in La_3_Cu_3_Bi_4_ and La_3_Cu_3_Sb_4_, due to the relatively large atomic number of Bi/Sb. Here relevant states of Bi/Sb are *p* states, which experience less electronic screening and hence a stronger nuclear charge, enhancing the effect of spin-orbit coupling. The band structures calculated without spin orbit coupling are also shown in Fig. 2(c,d) by red dashed line. We find that the magnitude of spin orbit splitting increases as we go from La_3_Cu_3_P_4_ to La_3_Cu_3_Bi_4_. This is expected, as spin orbit Hamiltonian is stronger for larger atomic numbers. As can be seen from Fig. 2(c,d), the spin-orbit interaction not only lifts the degeneracy of the bands but also increases the splitting of bands with increasing atomic number of the X atom.

In principle, materials with complex band structures and higher band degeneracies lead to better thermoelectric performance than single parabolic bands. This can happen when multiple bands have their band extrema with little or no difference in energy (orbital degeneracy or “band convergence”), or when multiple carrier pockets in the Brillouin zone are degenerate because they are symmetrically equivalent due to the symmetry of the crystal (valley degeneracy). Generally for cubic compounds such as the ones studied here, the symmetry results in threefold degenerate *p* orbitals at Γ point because of the equivalency of the *x*, *y*, and *z* directions in the Brillouin zone. However, due to spin orbit coupling the *p*_*z*_ orbital splits from the *p*_*x*_ and *p*_*y*_ orbitals, where the magnitude of the splitting is determined by the strength of spin orbit coupling. As a result, the valence bands at the Γ point split into a doubly degenerate band and a nondegenerate band, mainly composed of *p*_*x*/*y*_ and *p*_*z*_ orbitals, respectively which originate from atom X. As shown in the band structures due to the low atomic mass the splitting is minimal (37 meV) for La_3_Cu_3_P_4_ and La_3_Cu_3_As_4_. Also the dispersion of these first three bands near VBM is dramatically different; two bands being relatively heavy and one band being highly dispersive (nearly parabolic). Usually the lower splitting and a combination of heavy and light bands results in higher thermoelectric performance^14,22,23,57^. This suggests that the thermoelectric performance of La_3_Cu_3_P_4_ will be higher than that of the other compounds. On the other band the VBM for La_3_Cu_3_Bi_4_, and La_3_Cu_3_Sb_4_ is located slightly off the Γ along Γ-H direction resulting in no band degeneracy. Furthermore, other subsidiary maxima is located at ~80 meV below the VBM along Γ-N direction. For the most part all compounds show a considerable asymmetry between the valence and conduction bands, and therefore will behave differently under hole and electron doping. The band forming the conduction band edge is more dispersive than the valence band edge. Thus, the effective mass of the holes is much larger than that of the electrons as shown in Table 1, indicating that *p*-doping will have higher thermopower than *n*-doping. The overall electronic structure of these materials is quite similar and as discussed in the next section the main factor affecting the electronic transport in these compounds is the magnitude of bandgap not the topology of the bands.

### Thermopower and power factor

**Figure 4:**
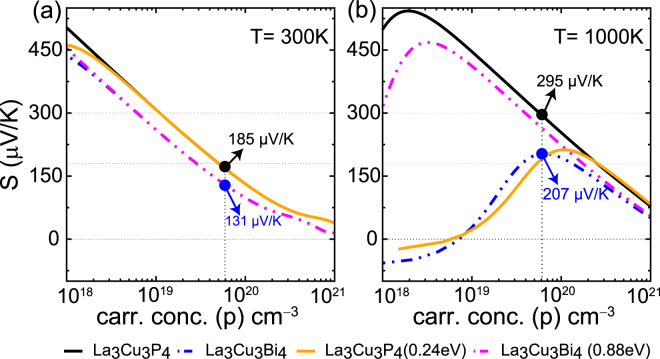
Calculated thermopower at (**a**) 300 K, and (**b**) 1000 K for La_3_Cu_3_P_4_, and La_3_Cu_3_Bi_4_. The brown dashed lines represents the limitation of thermopower (in the range of 180–300 *μ*V/K) for good thermoelectric materials. For comparison thermopower under *p*-type doping is also highlighted for both the compounds at carrier concentration of 6 × 10^19^ cm^−3^. *p*-type thermopower for La_3_Cu_3_P_4_ with the bandgap of La_3_Cu_3_Bi_4_ (solid orange line) and *vice versa* for La_3_Cu_3_Bi_4_ (dashed magenta lines) are also shown for comparison. Here the bandgap is modulated by applying scissor operator within the BoltzTraP code.

For a given value of carrier concentration and relaxation time, we can calculate the thermopower and electrical conductivity using Boltzmann transport theory as introduced in the Methods section. In these calculations, the thermopower and electrical conductivity tensor are calculated by integrating over all the electronic states. Figure 4(a,b) show the *p*-type thermopower for La_3_Cu_3_P_4_, and La_3_Cu_3_Bi_4_ as a function of carrier concentration at 300 and 1000 K. The thermopower plots for La_3_Cu_3_As_4_, and La_3_Cu_3_Sb_4_ are given in the Supplementary Information Fig. S4. By using the Wiedemann-Franz relation, we can rewrite the expression of *ZT* as, $ZT=\frac{r{S}^{2}}{{L}_{0}}$. Here *r* = *κ*_*e*_/(*κ*_*e*_ + *κ*_*l*_) and L_0_ is the Lorenz number for semiconductor. This relation tells us that even if the contribution of lattice thermal conductivity to *ZT* is ignored (*r* = 1), a thermopower S = 156 *μV*/*K* is required to achieved a *ZT* of 1.

We discuss La_3_Cu_3_P_4_ and La_3_Cu_3_As_4_ first. The 300 K thermopower plots exhibits logarithmic dependence on carrier concentration, in agreement with Pisarenko behavior. As seen, high thermopower (>200 *μ*V/K) is obtained even at quite low temperatures (300 K) with doping concentration up to 5 × 10^19^ cm^−3^. At 1000 K for both La_3_Cu_3_P_4_ and La_3_Cu_3_As_4_ thermopowers in the range of 180–300 *μ*V/K are found for hole concentrations between 6 × 10^19^ cm^−3^ to 2 × 10^20^ cm^−3^. Due to the substantial bandgap of La_3_Cu_3_P_4_ and La_3_Cu_3_As_4_, there are no bipolar effects and therefore thermopower keeps on increasing with temperature even at low doping concentrations. Due to the higher effective masses (presented in Table 1) the thermopower for *p*-type doping is nearly 1.5 times higher than that of *n*-type doping. Thermopower under *n*-type doping is presented in the Supplementary Information Fig. S4. Next we discuss thermopower for La_3_Cu_3_Sb_4_ and La_3_Cu_3_Bi_4_. With a calculated bandgap of 0.24 and 0.33 eV for Bi and Sb compound we expect significant bipolar conduction at high temperatures. The experimental measurements for hole doped thermopower of La_3_Cu_3_Sb_4_ were carried out by Fess *et al* ^30^. Their measured 300 K thermopower of ~60 *μ*V/K at hole doping of 5.1 × 10^20^ cm^−3^ agrees well with our calculated value of 55 *μ*V/K. As shown in Fig. 4(b) we find that 1000 K hole doped thermopower barely exceeds 200 *μ*V/K. The *n*-type thermopower does not even exceed −180 *μ*V/K (shown in Supplementry Information Fig. S4.), indicating poor thermoelectric performance under *n*-type doping.

As explained before the main  advantage of P and As compounds is their higher bandgap and density of states mass, which gives rise to large thermopower. The thermopower of La_3_Cu_3_P_4_ and La_3_Cu_3_Bi_4_ is compared in Fig. 4 and thermopower at hole concentration of 6 × 10^19^ cm^−3^ is also marked for comparison. As can be seen in Fig. 4(a), at 300 K *p*-type thermopower of La_3_Cu_3_P_4_ is 1.4 times higher than that of Bi compounds. Since the bipolar effect does not come into play at room temperature the origin of this thermopower should lie in the electronic structure. As discussed before the hole effective mass is ~1.8 times higher in La_3_Cu_3_P_4_ than in La_3_Cu_3_Bi_4_, which contributes to its high thermopower. As we go toward higher temperatures (shown for 1000 K) bipolar effect starts to contribute for La_3_Cu_3_Bi_4_ which reduces its thermopower at carrier concentration below 6 × 10^19^ cm^−3^. In order to quantify the gain in thermopower from the enhanced bandgap we performed thermopower calculation for La_3_Cu_3_P_4_ with the bandgap of La_3_Cu_3_Bi_4_ (0.24 eV) and *vice versa* for La_3_Cu_3_Bi_4_. Here the bandgap was modified by applying the scissor operator within the BoltzTraP code. These results are shown in Fig. 4(a,b). As expected at room temperature for most of the carrier concentration range the thermopower obtained with the modified bandgap overlaps with the one obtained with the original bandgap. Situation is different at 1000 K. The thermopower for La_3_Cu_3_P_4_ with 0.24 eV bandgap approaches same value as the La_3_Cu_3_Bi_4_ themopower at carrier concentrations lower than 6 × 10^19^ cm^−3^ due to enhanced bipolar effects. However at higher carrier concentrations in particular past 2 × 10^20^ cm^−3^ the La_3_Cu_3_P_4_ thermopower merges with the original curve. This is because, at high carrier concentrations higher effective mass of La_3_Cu_3_P_4_ enhances its thermopower. Similarly the thermopower for La_3_Cu_3_Bi_4_ with bandgap of 0.88 eV approaches the La_3_Cu_3_P_4_ thermopower at low carrier concentrations. However, due to its low effective mass at high carrier concentrations, thermopower remains more or less same as the original La_3_Cu_3_Bi_4_ thermopower. This analysis shows that the higher bandgap along with the high effective mass contributes to the higher thermopower of La_3_Cu_3_P_4_ when compared with the smaller bandgap compound La_3_Cu_3_Bi_4_.

**Figure 5:**
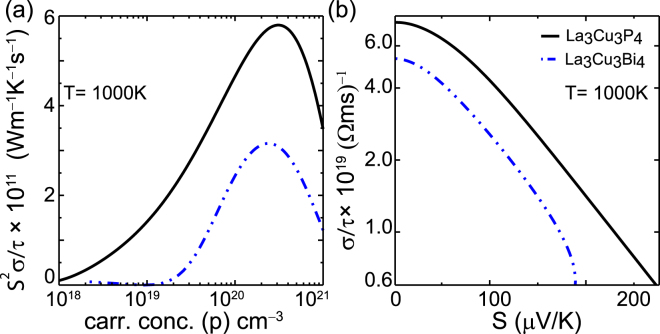
(**a**) Calculated power factor with respect to relaxation time $(\tfrac{{S}^{2}\sigma }{\tau })$ at 1000 K as a function of *p*-type doping for La_3_Cu_3_P_4_, and La_3_Cu_3_Bi_4_. (**b**) *σ*/*τ vs* thermopower under *p*-type doping for La_3_Cu_3_P_4_, and La_3_Cu_3_Bi_4_ at 1000 K. Note that y-axis in part (b) is in log scale.

In order to present more information on thermoelectric behavior, we calculate the power factor divided by the inverse scattering rate (*S* ^2^ *σ*/*τ*), at 1000 K, as illustrated in Fig. 5(a) for La_3_Cu_3_P_4_ and La_3_Cu_3_Bi_4_. The power factor for La_3_Cu_3_As_4_ and La_3_Cu_3_Sb_4_ can be found in the Supplementary Information Fig. S5. Also as these materials show promising thermopowers under *p*-type doping from this point onward mainly the results for hole doping will be discussed. Both La_3_Cu_3_P_4_ and La_3_Cu_3_As_4_ show relatively higher power factor than that of La_3_Cu_3_Sb_4_ and La_3_Cu_3_Bi_4_. To explain the high power factor for P and As compounds we plot the thermopower and *σ*/*τ* for La_3_Cu_3_P_4_ and La_3_Cu_3_Bi_4_ at 1000 K under *p*-type doping as shown in Fig. 5(b). In the typical required thermopower range for high performance thermoelectric materials (180–200 *μ*V/K), the *σ*/*τ* of La_3_Cu_3_P_4_ exceeds *σ*/*τ* of La_3_Cu_3_Bi_4_ by more than a factor of 2.4. If the scattering time for these two compounds assumed to be the same, the electric conductivity of La_3_Cu_3_P_4_ should be higher than La_3_Cu_3_Bi_4_. The relatively high *σ*/*τ* value for La_3_Cu_3_P_4_ in comparison with La_3_Cu_3_Bi_4_ is bit surprising, as hole effective mass of former is significantly larger than the latter as shown in Table 1. For P and As compounds two weak dispersive bands degenerated at the Γ point form heavier bands, which leads to a large thermopower. Another strong dispersive band is approximately parabolic near the Γ point, which is helpful to promote its carrier mobility. Thus, the combination of these heavy and light bands in the upper valence band is responsible for the relatively large thermopower and *σ*/*τ* in La_3_Cu_3_P_4_ and La_3_Cu_3_As_4_.

Within the framework of Boltzmann transport theory the electrical conductivity is calculated by assuming the constant relaxation time. However for a real estimate of electrical conductivity it is necessary to have information about relaxation time *τ*. Here, we model *τ* by comparing the calculated *σ*/*τ* to the experimentally measured electrical conductivity. Specifically, for *p*-type La_3_Cu_3_Sb_4_, the room temperature electrical conductivity data from Fess *et al*.^30^ at a carrier concentration of 5.13 × 10^20^ cm^−3^ was used. At this doping level reported electrical conductivity is approximately 3.7 × 10^4^ S/m, which yields a *τ* value of 3.3 × 10^−15^ s. At last since the electronic structure near the band edge is similar for all 4 compounds, we use the same relaxation time *τ* for *ZT* calculations in all four compounds.

The lattice thermal conductivity (*κ*_*latt*_) of these compounds was calculated in our recent work^32^, where a low *κ*_*latt*_ of 1W/mK was found at high temperatures (>900 K) for all four compounds. The 300 K *κ*_*latt*_ for all four compounds are reproduced in Table 1 for comparison. The *κ*_*latt*_ for La_3_Cu_3_Sb_4_ and La_3_Cu_3_Bi_4_ is in the range with the measured values for the other homologous compounds. For instance at 300 K a *κ*_*latt*_ of 3.8, 1.6, and 2.5 W/mK has been reported for Y_3_Cu_3_Sb_4_^58^, Ce_3_Cu_3_Sb_4_^30^, and La_3_Cu_3_Sb_4_^30^. As discussed in ref.^32^, the *κ*_*latt*_ for P and As compounds is comparable with the state of the art thermoelectric materials such as PbTe^59^ and Bi_2_Te_3_^60,61^, indicating their potential for a good thermoelectric performance. This is counter-intuitive that despite of their lower atomic masses P and As exhibit lower *κ*_*latt*_; as in general compounds with heavier atoms results in low value of *κ*_*latt*_. The origin of this abnormal behavior in *κ*_*latt*_ for P and As compounds have been discussed in ref.^32^. Another important parameter which governs the figure of merit is the electronic contribution to the thermal conductivity (*κ*_*e*_). Here *κ*_*e*_ is calculated by Wiedemann–Franz law.

### Figure of merit

**Figure 6:**
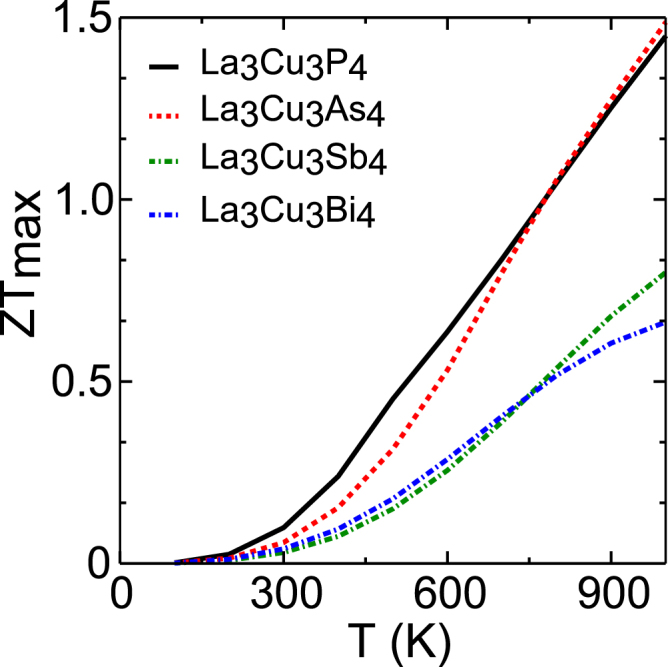
Calculated figure of merit as function of temperature for La_3_Cu_3_P_4_, La_3_Cu_3_As_4_, La_3_Cu_3_Sb_4_, and La_3_Cu_3_Bi_4_ under *p*-type doping. For *ZT*_*max*_ calculation *ZT* is calculated as a function of carrier concentration at temperatures 100 K to 1000 K, and maximum value is shown in the plot.

Finally we model *ZT* as a function of the temperature as shown in Fig. 6 for *p*-type doping. For *ZT*_*max*_ calculation, *ZT* is calculated as a function of carrier concentration at temperatures ranging from 100 K to 1000 K, and maximum value is shown in the plot. The maximum value of *ZT* increases with increasing doping levels and temperatures. A peak *ZT* of ~1.5 at 1000 K is found with a doping level of 7 × 10^20^ cm^−3^ for *p*-type doped La_3_Cu_3_P_4_ and La_3_Cu_3_As_4_. The *ZT* under *n*-type doping is presented in the Supplementary Information Fig. S6. Under *n*-type doping due to low power-factor (thermopower) performance is rather poorer than that of *p*-type and a *ZT* of 0.75 was achieved for La_3_Cu_3_P_4_ and La_3_Cu_3_As_4_. A lower *ZT* of 0.7 and 0.45 was achieved for La_3_Cu_3_Sb_4_/La_3_Cu_3_Bi_4_ under *p*-type and *n*-type doping due to their relatively lower thermopower/electrical conductivity and higher *κ*_*latt*_. It is noteworthy that experimental values of *ZT* will depend on actual scattering times, so the values reported here should be taken only as first estimates. None of the less, our results indicate the potential good thermoelectric performance of *p*-type La_3_Cu_3_P_4_ and La_3_Cu_3_As_4_ at high temperatures (~1000 K). As the efficient performance is predicted at high temperatures one concern in this regard could be the melting point of these compounds. A rough estimate of melting point around 2120 K for La_3_Cu_3_Sb_4_ was made by Fess *et al*.^30^ by fitting the thermal expansion coefficient (*α*). However, no study is available on actual thermal stability of these compounds, hence we restrict our *ZT* analysis to a much lower temperature of 1000 K. Our calculations show that if the La_3_Cu_3_P_4_ and La_3_Cu_3_As_4_ compounds remain stable at higher temperatures such as 1500 K a much higher *ZT* of >2 can be achieved. Our calculated *ZT* values are comparable with other state of the art *p*-type thermoelectric materials such as skutterudites (*ZT* = 0.9 at 800 K)^62^, PbTe (*ZT* = 1.8 at 900 K)^63^, SnTe (*ZT* = 1.1 at 800 K)^64^, and FeNbSb (*ZT* = 1.5 at 1200 K)^65^. Due to the various approximations involved in the theoretical calculations a quantitative comparison of *ZT* with the experimental measurements is often elusive. To put our calculated *ZT* values in real perspective we compare these with recent theoretical studies, where similar methodologies were employed. For instance a *ZT* of 1.75, 0.8, 1.75, 0.9, and 1.9 has been reported for Mg_2_Ge_0.5_Sn_0.5_ (1000 K)^7^, Bi_2_S_3_ (750 K)^66^, BaCd_2_Sb_2_ (950 K)^67^, WS_2_ (1500 K)^68^ and Si_2_Te_3_ (1000 K)^69^, respectively. Thus, the calculated *ZT* values of La based compounds are comparable with the other theoretically reported values for a variety of materials.

## Discussion

We present structural, electronic and thermoelectric properties of virtually unexplored La_3_Cu_3_X_4_ compounds. By using global structure search method with the particle swarm optimization algorithm we conclude that, the structure of La_3_Cu_3_P_4_ and La_3_Cu_3_As_4_ will be the same as other homologous compounds *i*.*e*. La_3_Cu_3_Sb_4_ and La_3_Cu_3_Bi_4_. The electronic structure calculations reveal that all four compounds studied here are semiconducting with the bandgaps in the range of 0.24 to 0.88 eV. This large bandgap for P and As compounds, while maintaining the high electrical conductivity results in nearly ~2 fold enhancement in thermopower when compared to the Sb and Bi compounds. Our results make clear that the La based ternary rare earth compounds studied here are quite likely to have high power factor, which together with low *κ*_*latt*_ as discussed in ref.^32^ results in high figure of merit. In particular we report that a *ZT* of 1.5 can be achieved for La_3_Cu_3_P_4_ and La_3_Cu_3_As_4_ under *p*-type doping at a doping level of 7 × 10^20^ cm^−3^. Assuming that the relaxation time (*τ*) does not vary significantly among these compounds, we compare the 300 K calculated figure of merit with other homologous compounds, such as Y_3_Cu_3_Sb_4_, Ce_3_Cu_3_Sb_4_, and La_3_Cu_3_Sb_4_. All these compounds are narrow bandgap semiconductor with the bandgap in the range of 0.2–0.25 eV, and suffer from low figure of merit, mainly due to low thermopower (~50–100 *μ*V/K at 300 K). For example at room temperature *ZT* values of 0.01, 0.03, and 0.015 have been reported for Y_3_Cu_3_Sb_4_^58^, Ce_3_Cu_3_Sb_4_^30^, and La_3_Cu_3_Sb_4_^30^, respectively. Our calculated 300 K *ZT* value of 0.018 for La_3_Cu_3_Sb_4_ is in good agreement with experimentally measured value of 0.015^30^. It is noteworthy that all the previous *ZT* measurements for these compounds are limited to room temperature where efficient performance is unlikely. Even with the low thermal conductivity, our calculation for hole doped P and As compounds find a small *ZT* of 0.1 and 0.06 at room temperature. The origin of enhanced figure of merit for P and As compounds at high temperatures is related to (i) their relatively larger bandgap which results in large thermopower (powerfactor) at elevated temperatures and (ii) their low thermal conductivity (as discussed in ref.^32^). Our study demonstrates that if synthesized experimentally La_3_Cu_3_P_4_ and La_3_Cu_3_As_4_ can be potential high performance thermoelectric materials. For experimentally known and explored compounds (La_3_Cu_3_Sb_4_ and La_3_Cu_3_Bi_4_) our results imply that the measurements at high temperatures are required where better thermoelectric performance is anticipated. We hope that our current findings will stimulate future experimental exploration of the thermoelectric properties of these and other similar compounds.

## Methods

In order to predict the structure of La_3_Cu_3_P_4_ and La_3_Cu_3_As_4_ we use a global structure search method with the particle swarm optimization (PSO)^41^ algorithm as implemented in the Calypso code^43^. For structure prediction calculations the number of La_3_Cu_3_P_4_/La_3_Cu_3_As_4_ formula units per cell was allowed to vary from one (10 atoms) to four (40 atoms), and no restriction was placed on the space group. The number of structures in each generation was set to 40 and the total number of generations was 15 during the structure evolution. The structures generated by Calypso at each generation are then optimized to local minima and subsequently the total energies are calculated by using the VASP code^70–72^. For VASP calculations, we employ the projector augmented wave type pseudopotential and the Perdew Burke Ernzerhof (PBE) version of the exchange-correlation functional^70–72^. A 500 eV kinetic energy cutoff and Γ-centered k-mesh with k-spacing of 0.4/Å were used for structure relaxation and a denser k-mesh with k-spacing of 0.15/Å for total energy calculation. During structure relaxation, both lattice constants and atom coordinates are optimized until the forces on each atom were smaller than 0.005 eV/Å.

The electronic structure calculations were performed within density functional theory using the linearized augmented plane-wave (LAPW) method with local orbitals^73,74^ as implemented in the WIEN2K code^75^. The LAPW sphere radii were set to 2.5, 2.5, and 2.2 for La, Cu, and X, respectively. In addition R*k*_*max*_ = 9.0, was used to ensure the well convergence of basis set, where R and *k*_*max*_ are the smallest LAPW sphere radius and interstitial plane-wave cutoff, respectively. The Brillouin zone was sampled by taking 5000 **k**-points for all crystal structures. In order to obtain an accurate bandgap, we employed a modified Becke-Johnson functional of Tran and Blaha (TB-mBJ)^76,77^. Additionally, spin orbit coupling was incorporated to account for relativistic effects. The computed electronic structures with the TB-mBJ functional were also used to obtain transport coefficients with 30000 reducible **k**-points. The transport calculations were done by solving Boltzmann transport equation^78^ within the constant scattering time approximation (CSTA)^78,79^ as implemented in the BoltzTraP code^80^.

## Electronic supplementary material

Supplementary Information
