# Supplementary material for: High potential thermoelectric figure of merit in ternary La3Cu3X4 (X = P, As, Sb and Bi) compounds
Source: Sci Rep. 2017 Oct 27;7:14270. doi: 10.1038/s41598-017-14658-5 (PMC5660201; doi:10.1038/s41598-017-14658-5)
Supplement: Supplementary file 1 — Supplementary Information [file 41598_2017_14658_MOESM1_ESM.pdf]

Supplementary Information:

High potential thermoelectric figure of merit in ternary $La_3Cu_3X_4$ (X = P, As, Sb and Bi) compounds

Tribhuwan Pandey*and David S. Parker†

Material Science and Technology Division, Oak Ridge National Laboratory, Oak Ridge, Tennessee 37831, USA

Phonon dispersion for $La_3Cu_3P_4$ in space group $Ia3$

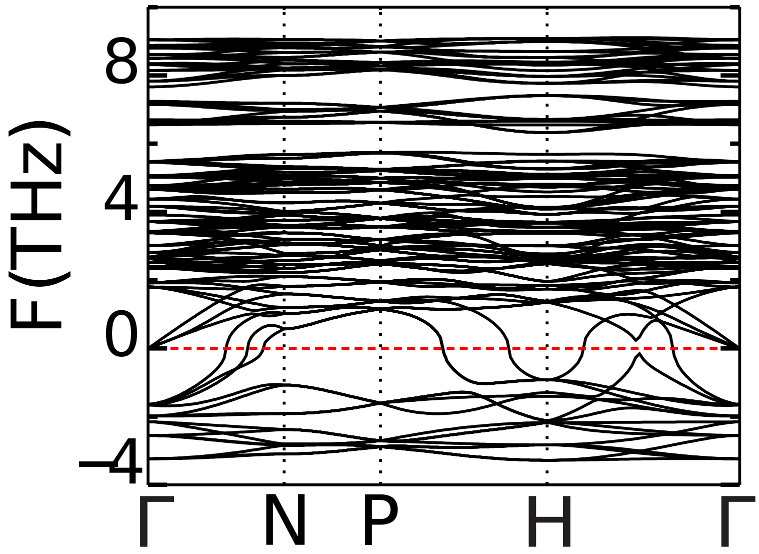

Figure S1: The calculated phonon dispersion for $La_3Cu_3P_4$ in space group $Ia3$. The phonon dispersion exhibits negative frequencies indicating the dynamical instability of this structure.

Stability analysis of $La_3Cu_3P_4$ and $La_3Cu_3As_4$

As discussed before the $La_3Cu_3P_4$ and $La_3Cu_3As_4$ stoichiometric phases for La-Cu-P and La-Cu-As are not yet reported. In order to establish the thermodynamic stability of these phases we list there the formation enthalpy by the Materials Project [1, 2, 3] and AFLOWLIB data base [4, 5] as well as the compounds reported experimentally as shown in Table S1. Considering La-Cu-P first, all the binary compounds have formation enthalpies substantially smaller in magnitude than $La_3Cu_3P_4$, so that most potential decomposition pathways are energetically blocked. The exception of this are LaP and LaP_2, which shows a ΔH_f of -1.5 eV/atom and -1.1 eV/atom, respectively. Next we used the grand-canonical linear programming (GCLP) method [6, 7] to determine the lowest-free-energy thermodynamic decomposition reactions pathways for La-Cu-P and La-Cu-As systems. GCLP is an automated method to determine the thermodynamically preferred reaction pathways.
We first consider the GCLP calculations of $La_3Cu_3P_4$ decomposition, by using the T = 0 K DFT energies without including

*pandeyt@ornl.gov
†parkerds@ornl.gov

the zero point energy and identify the lowest-energy thermodynamic decomposition pathway. We find that the thermodynamic decomposition of $La_3Cu_3P_4$ into other stable binary phases such as LaP and LaP_3 can be best described by the following set of equations:

$La_3Cu_4P_4$
$\longrightarrow x_1$LaP + $x_2La_5Cu_5B_{12}$ + x_3Cu − 137.2 meV/atom
$\longrightarrow x_1$LaP + x_2CuP_2 + x_3Cu − 192.4 meV/atom
$\longrightarrow x_1$LaP + x_2LaP_2 + x_3Cu − 165.6 meV/atom
$\longrightarrow x_1$LaP + x_2LaP_5 + x_3Cu − 175.9 meV/atom

Similarly, decomposition of $La_3Cu_3As_4$ into its stable binary phases such as LaAs, $LaAs_2$ and La_4As_3 can be represented by:

$La_3Cu_4As_4$
$\longrightarrow x_1$LaAs + x_2LaAs_2 + x_3Cu − 151.3 meV/atom
$\longrightarrow x_1La_4As_3$ + x_2LaAs_2 + x_3Cu − 56.1 meV/atom
$\longrightarrow x_1La_2CuAs$ + x_2LaAs_3 + x_3Cu − 18.3 meV/atom
$\longrightarrow x_1$LaCuAs + x_2LaAs_3 + x_3Cu − 84.3 meV/atom

Here the x_i coefficients that minimize the right hand side of this equation are determined subject to the stochiometry constraints; $\sum_i x_i = 3$. It is notable that for all the decomposition pathways discussed here the energy gain is less than 190 meV/atom. The discussed decomposition of $La_3Cu_3P_4$ and $La_3Cu_3As_4$ into the more favorable binary phases is still a possibility, however compounds with similarly calculated energetics do in fact form. For example regardless of their much lower formation enthapies, $La_5Cu_{19}P_{12}$ (ΔH_f = −0.542) [8], and $LaCu_4P_3$ (ΔH_f = −0.85) [9] are already reported to be stable experimentally. These calculations thus suggest that $La_3Cu_3P_4$ and $La_3Cu_3As_4$ *may* form, and leave the matter for experimental inquiry.

Table S1: Formation enthalpies (ΔH_f) per atom of La-Cu-P and La-Cu-As systems for all phases as given by the Materials project [1, 2, 3] and **AFLOWLIB** [4, 5] data base. The ΔH_f for our reported structures is also listed for comparison. Here superscripts ‡ represents experimentally reported compounds, * represents compounds from Materials project data base, $^\triangle$ denotes compounds from AFLOWLIB data base, and $^\intercal$ represents compounds calculated in this work. Note that few compounds repeat for both La-Cu-P and La-Cu-As systems, as they are possible phases for both of them.

La-Cu-P		La-Cu-As	
System	ΔH_f/atom (eV)	System	ΔH_f/atom (eV)
$Cu_2P_7^*$	−0.105	$LaAs^*$	−1.572
CuP_{10}^*	−0.048	$LaAs_2^*$	−1.062
CuP_2^*	−0.117	$LaCu_2^*$	−0.218
$LaCu_2^*$	−0.218	$LaCu_6^*$	−0.135
$LaCu_5^*$	−0.152	Cu_2As^*	0.02
$LaCu_6^*$	−0.135	Cu_3As^*	0.079
LaP^*	−1.51	$Cu_3As_4^*$	0.264
LaP_2^*	−1.102	$Cu_5As_2^*$	0.05
LaP_5^*	−0.573	$CuAs^*$	0.052
LaP_7^*	−0.437	$CuAs_2^*$	0.081
Cu_3P^*	−0.022	$La_4As_3^*$	−1.339
$LaCu_4^*$	−0.161	$LaCu_4^*$	−0.161
$LaCu_6^*$	−0.129	$LaCu_6^*$	−0.129
LaP_3^*	−0.397	$LaCu_4As^\triangle$	−0.17
$LaCu_4P_3^\ddagger$	−0.85	$LaCuAs^\triangle$	−0.92
$La_5Cu_{19}P_{12}^\ddagger$	-0.542	$LaCu_5^*$	−0.152
$La_4CuP^\triangle$	0.422	$La_2CuAs^\triangle$	−0.69
$La_4Cu_4P_2^\triangle$	−0.44	$LaCu_2As^\triangle$	−0.102
$La_2Cu_4P_4^\triangle$	−0.76	$\mathbf{La_3Cu_3As_4^\intercal}$	**−0.90**
$LaCu_2P^\triangle$	−0.04		
$LaCuP^\triangle$	−0.53		
$La_2CuP^\triangle$	−0.03		
$La_4Cu_4P_8^\triangle$	−0.84		
$LaCuP_2^\triangle$	−0.33		
$\mathbf{La_3Cu_3P_4^\intercal}$	**−0.95**		

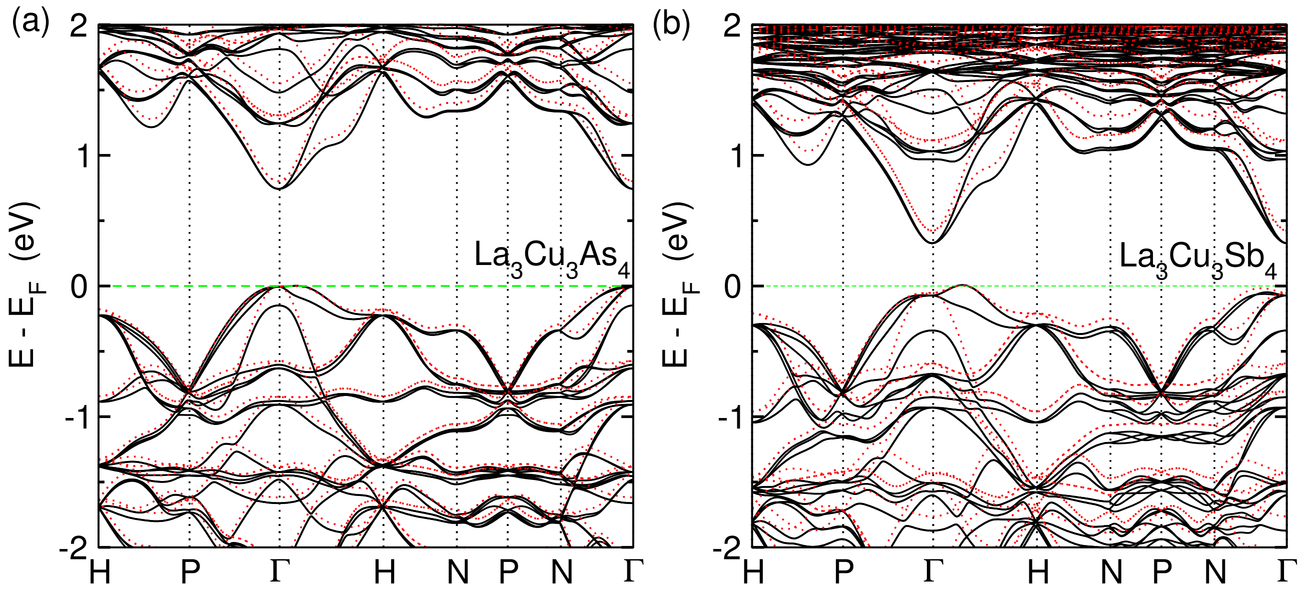

Figure S2: Band structures as calculated with TB-mBJ functional for (a) $La_3Cu_3As_4$, and (b) $La_3Cu_3Sb_4$. The valence band maxima is referenced to 0 eV. The solid black lines represent band structure with spin orbit coupling and dotted red lines represent band structure without spin orbit coupling.

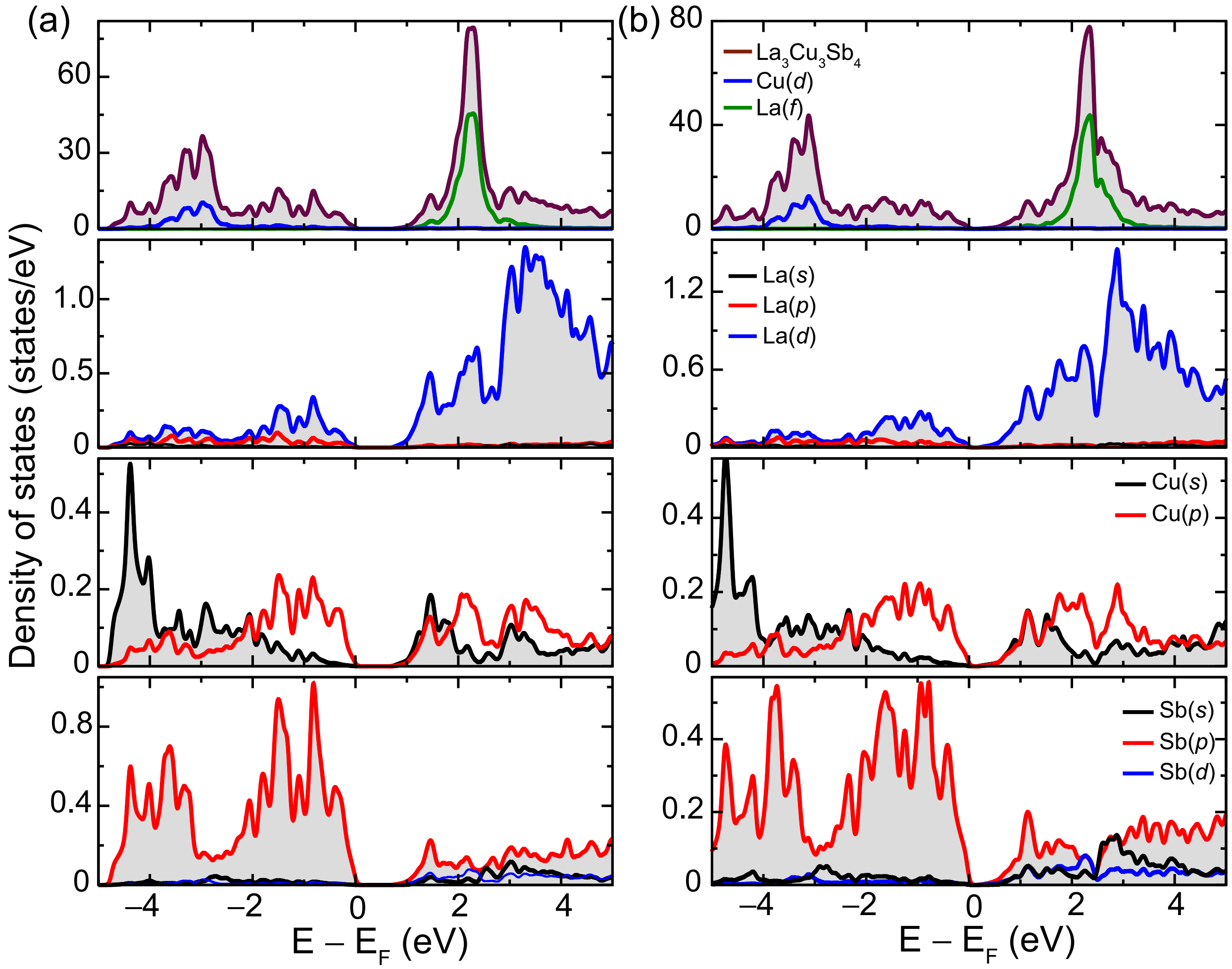

Figure S3: Total and partial density of states obtained using the TB-mBJ functional for (a) $La_3Cu_3As_4$, and (b) $La_3Cu_3Sb_4$. The valence band maxima is referenced to 0 eV. Legends in (a) are same as (b) where As color is same as Sb.

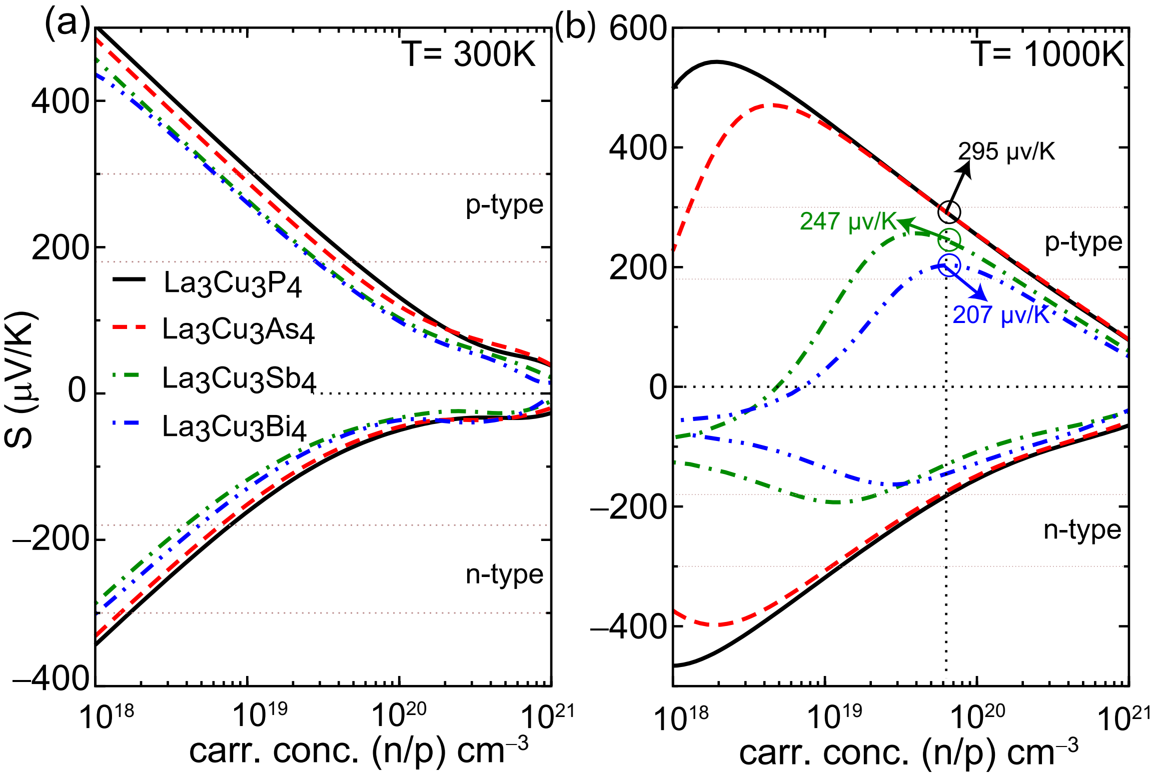

Figure S4: Calculated thermopower at (a) 300 K, and (b) 1000 K for $La_3Cu_3P_4$, $La_3Cu_3As_4$, $La_3Cu_3Sb_4$, and $La_3Cu_3Bi_4$. The brown dashed lines represents the limitation of thermopower (in the range of 180-300 μV/K) for good thermoelectric materials. For comparison thermopower under p-type doping is also highlighted at carrier concentration of 6×10^{19} cm^{-3}.

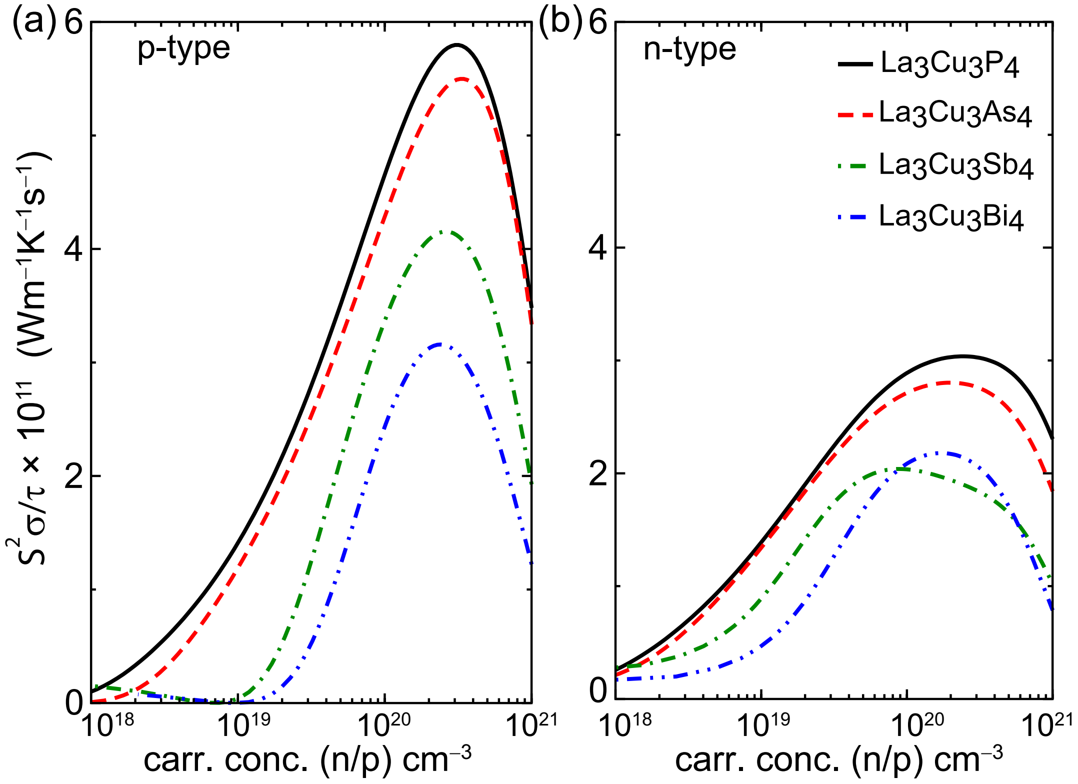

Figure S5: Calculated power factor at 1000 K with respect to relaxation time ($\frac{S^2\sigma}{\tau}$) at various temperature as a function of carrier concentration under (a) p-type doping, and (b) n-type doping for $La_3Cu_3P_4$, $La_3Cu_3Sb_4$, $La_3Cu_3Sb_4$, and $La_3Cu_3Bi_4$.

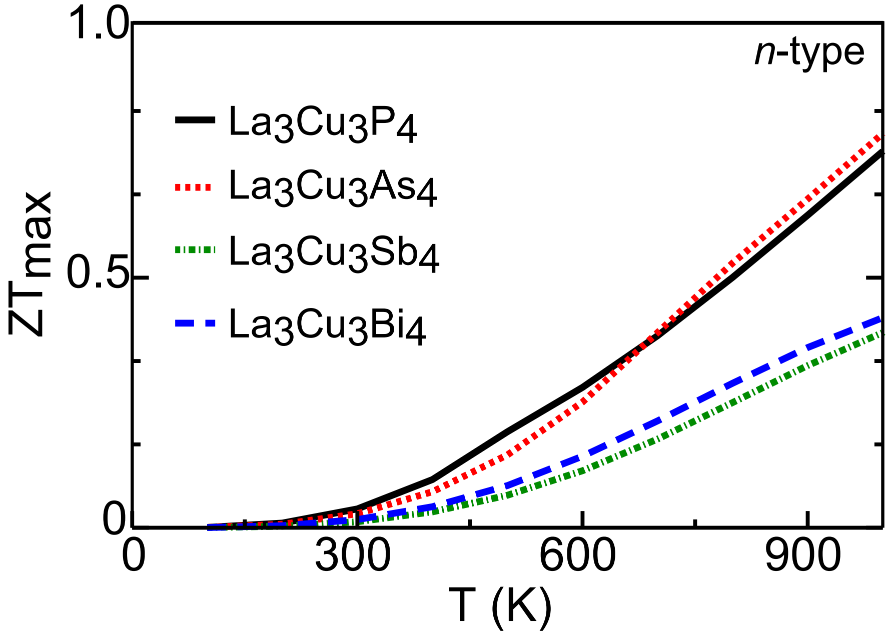

Figure S6: Calculated figure of merit as function of temperature for $La_3Cu_3P_4$, $La_3Cu_3As_4$, $La_3Cu_3Sb_4$, and $La_3Cu_3Bi_4$ under n-type doping. For ZT_{max} calculation ZT is calculated as a function of carrier concentration at temperatures 100 K to 1000 K, and maximum value is shown in the plot.

References

[1] A. Jain, S. P. Ong, G. Hautier, W. Chen, W. D. Richards, S. Dacek, S. Cholia, D. Gunter, D. Skinner, G. Ceder, K. a. Persson, The Materials Project: A materials genome approach to accelerating materials innovation, APL Mater. (1) 011002.

[2] S. P. Ong, L. Wang, B. Kang, G. Ceder, Li-Fe-P-O_2 phase diagram from first principles calculations, Chem. Mater. 20 (5) (2008) 1798–1807.

[3] A. Jain, G. Hautier, S. Ong, C. Moore, C. Fischer, K. Persson, G. Ceder, Formation enthalpies by mixing GGA and GGA+U calculations, Phys. Rev. B 84 (4) (2011) 045115.

[4] S. Curtarolo, W. Setyawan, S. Wang, J. Xue, K. Yang, R. H. Taylor, L. J. Nelson, G. L. Hart, S. Sanvito, M. Buongiorno-Nardelli, et al., AFLOWLIB. ORG: A distributed materials properties repository from high-throughput ab initio calculations, Comp. Mater. Sci. 58 (2012) 227–235.

[5] S. Curtarolo, W. Setyawan, G. L. Hart, M. Jahnatek, R. V. Chepulskii, R. H. Taylor, S. Wang, J. Xue, K. Yang, O. Levy, et al., AFLOW: an automatic framework for high-throughput materials discovery, Comp. Mater. Sci. 58 (2012) 218–226.

[6] A. R Akbarzadeh, V. Ozoliņš, C. Wolverton, First-principles determination of multicomponent hydride phase diagrams: Application to the Li-Mg-N-H system, Adv. Mater. 19 (20) (2007) 3233–3239.

[7] Y. Wang, Y. Zhang, C. Wolverton, First-principles studies of phase stability and crystal structures in Li-Zn mixed-metal borohydrides, Phys. Rev. B 88 (2) (2013) 024119.

[8] R. Cava, T. Siegrist, S. Carter, J. Krajewski, W. Peck Jr, H. Zandbergen, Crystal structure and elementary physical properties of $La_5Cu_{19}P_{12}$ and $Ce_5Cu_{19}P_{12}$, J. Solid State Chem. 121 (1) (1996) 51–55.

[9] J. Wang, K. Lee, K. Kovnir, Distorted phosphorus and copper square-planar layers in $LaCu_{1+x}P_2$ and $LaCu_4P_3$: Synthesis, crystal structure, and physical properties, Inorg. Chem. 54 (3) (2014) 890–897.
